# Supplementary material for: Realization of macroscopic ratchet effect based on nonperiodic and uneven potentials
Source: Sci Rep. 2021 Aug 16;11:16617. doi: 10.1038/s41598-021-96192-z (PMC8368205; doi:10.1038/s41598-021-96192-z)
Supplement: Supplementary file 1 — Supplementary Information. [file 41598_2021_96192_MOESM1_ESM.pdf]

SUPPLEMENTARY INFORMATION

Realization of macroscopic ratchet effect based on nonperiodic and uneven potentials.

V. Rollano,[1] A. Gomez,[2] A. Muñoz-Noval,[1,3] M. Velez,[4,5] M. C. de Ory,[1] M. Menghini,[1] E. M. Gonzalez,[1,3] and J. L. Vicent[1,3,a)]

[1] IMDEA-Nanociencia, Cantoblanco, E-28049 Madrid, Spain

[2] Centro de Astrobiología (CSIC-INTA), Torrejón de Ardoz, E-28850 Madrid, Spain

[3] Departamento Física de Materiales, Universidad Complutense, E-28040 Madrid, Spain

[4] Departamento de Física, Universidad de Oviedo, E-33007 Oviedo, Spain.

[5] CINN (Universidad de Oviedo-CSIC), E-33940 El Entrego, Spain.

a) E-mail: jlvicent@ucm.es

The magnetic configuration of the Co honeycomb lattice is described in terms of two kinds of −1/2 magnetic half vortices, either associated with a +1 magnetic charge (black half vortex) or with a −1 magnetic charge (white half vortex); each vertex contains two charged Néel walls.

The orientation of each half magnetic vortex in each vertex of the honeycomb array can be extracted taking into account the half magnetic vortex asymmetries and following the pseudo spin ice rules. That is: Two in - one out (+1 magnetic charge in the vertex) or two out - one in (-1 magnetic charge in the vertex) (1). In this way, we can have a picture of the whole sample with the +1 y -1 magnetic charge distribution.

Figures S1(a) - (c) depict: (a) MFM experimental image, (b) simulated MFM contrast and (c) simulated micromagnetic configuration. Comparison between (a-c) allows determining the position of ice charges +1 (black) and -1 (white) as sketched in Fig. 1 (d).

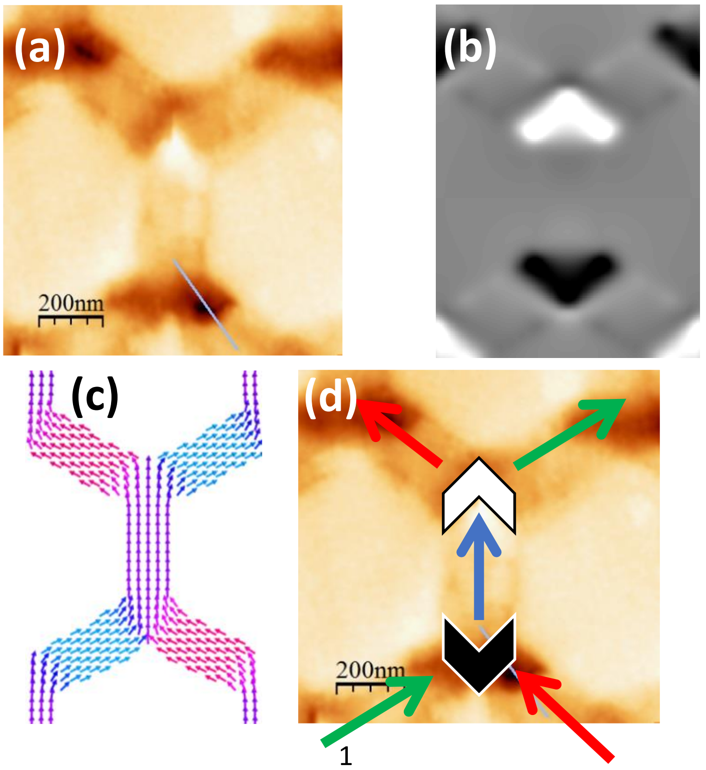

Figure S1. (a) Experimental image of a pair (-1, +1) of spin-ice charges (corresponding to magnetic half vortices) in a single bar of the array, (b) simulated MFM contrast and (c) simulated micromagnetic configuration, showing the two Néel walls in each vertex. Comparison between (a) and (c) allows determining the position of magnetic half vortices and the local magnetization orientation at each intersection as sketched in (d).

Figure S2 shows typical experimental MFM image of the honeycomb array in spin-ice I configuration. There is not any specific magnetic charge pattern in the picture.

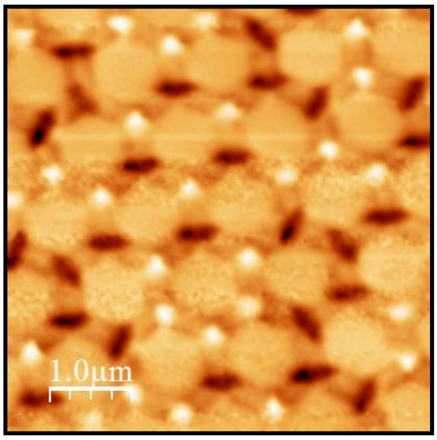

Figure S2 Experimental Magnetic Force Microscopy (MFM) image of the honeycomb array in spin-ice I configuration.

Following the pseudo spin ice rules, the magnetization directions can be known in the honeycomb bars. Figure 3 depicts the outcome.

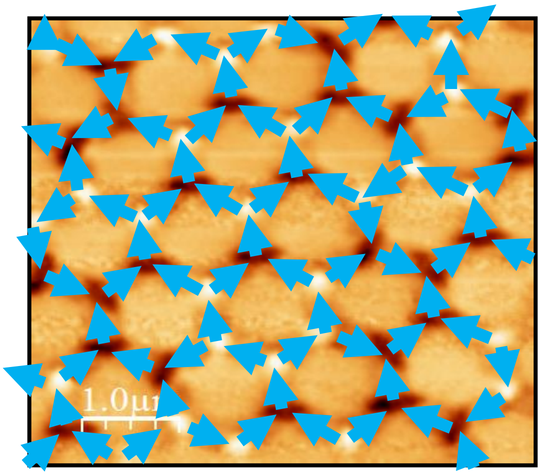

Figure S3. Magnetic dipole directions after the pseudo spin ice rules are applied to Figure S2 (MFM experimental image).

Worth to note that the V-shaped pairs of charged Néel domain walls of these two half vortices point in different directions along the honeycomb array in this spin ice I configuration; see

Figure S4. In this Figure the black V-shaped corresponds to +1 magnetic charge and the white V-shaped corresponds to -1 magnetic charge.

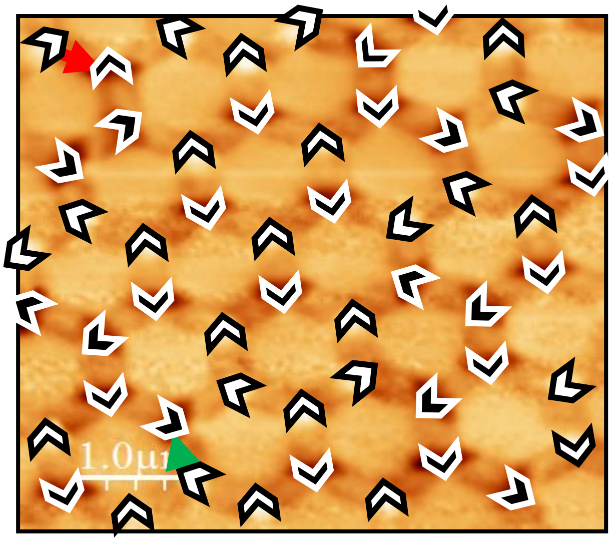

Figure S4. Distribution and orientation of the +1 (black) and – 1 (white) magnetic charge.

Therefore, a vortex on the move can cross different orientations of the two charged Néel walls, following the same trajectory.

In addition, this analysis allows a rough estimation of the direction of the remanent magnetization in both samples. Figure S5 shows a sketch.

Sample A:

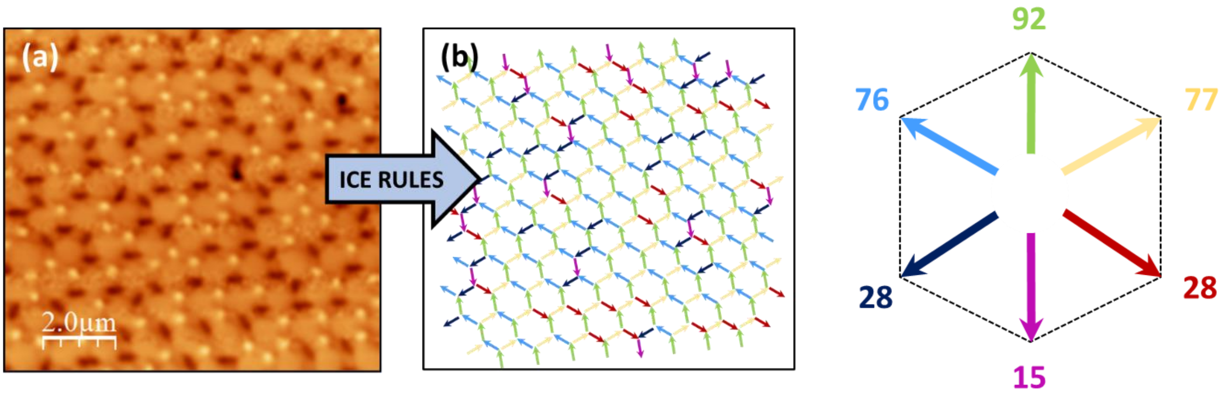

Sample B

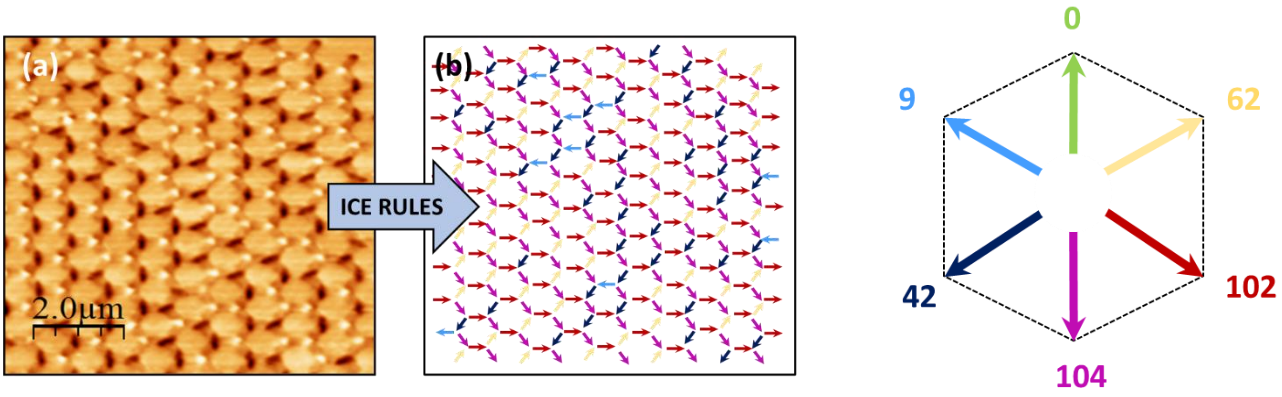

Figure S5. (a) Sample A. Magnetization distribution obtained from MFM experimental image following the ice rules. (b) Sample B. Magnetization distribution obtained from MFM experimental image following the ice rules.

Interestingly, in sample A the resultant total magnetization is pretty close to an easy axis direction, while in sample B the total magnetization is deviated from any symmetrical axes and with strength much higher than in sample A.

Figure S6 shows the difference between spin ice I (disordered) and spin ice II (ordered) configurations.

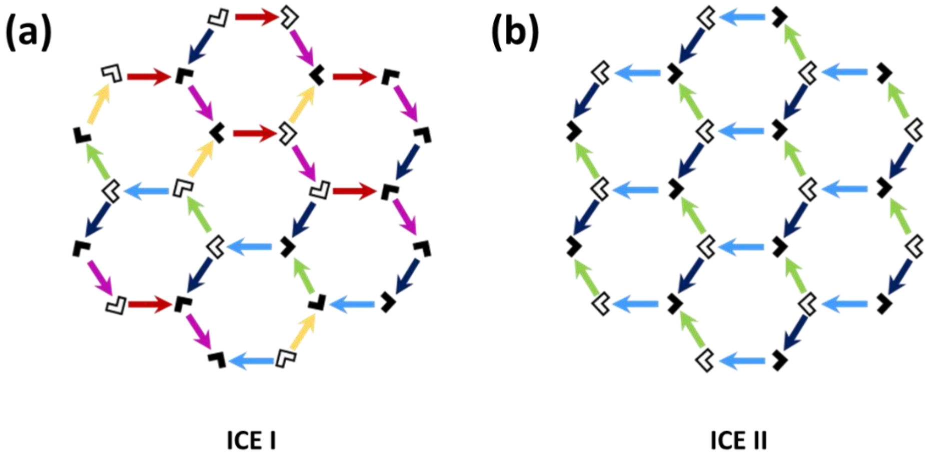

Figure S6. Examples of spin ice configurations. (a) Type I spin ice. (b) Type II spin ice.

Finally, in Figure S7 a sketch of the experiment geometry is depicted.

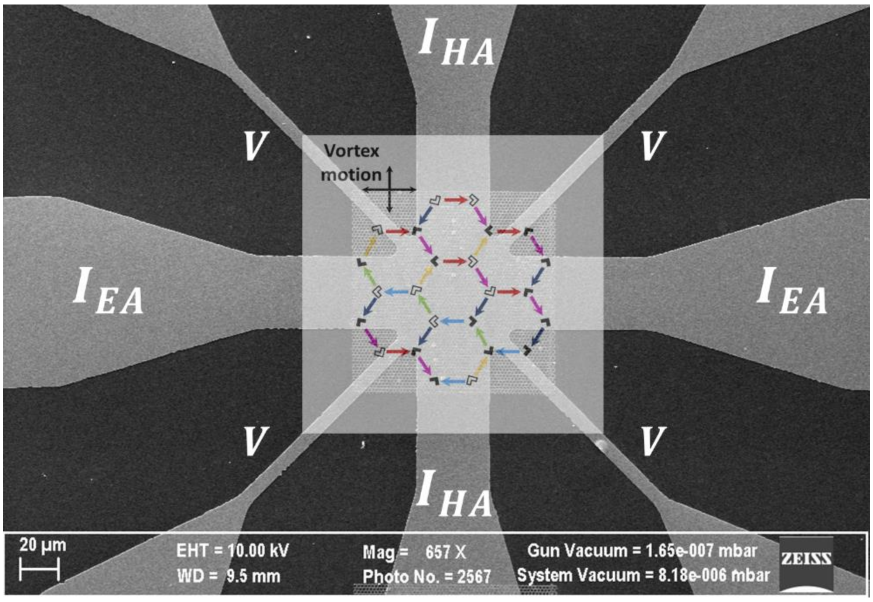

Figure S7. SEM image of the device. A drawing of the honeycomb array has been inserted. I_{EA} and I_{HA} indicate the current directions parallel to easy and hard axes respectively.

References

1 Tanaka, M., Saitoh, E., Miyajima, H., Yamaoka, T. & Iye Y. Magnetic interactions in a ferromagnetic honeycomb nanoscale network. *Phys. Rev. B* **73**, 052411 (2006)
